# Resting-state electroencephalographic characteristics related to mild cognitive impairments

**DOI:** 10.3389/fpsyt.2023.1231861

**Published:** 2023-09-13

**Authors:** Seong-Eun Kim, Chanwoo Shin, Junyeop Yim, Kyoungwon Seo, Hokyoung Ryu, Hojin Choi, Jinseok Park, Byoung-Kyong Min

**Affiliations:** ^1^Department of Applied Artificial Intelligence, Seoul National University of Science and Technology, Seoul, Republic of Korea; ^2^Department of Applied Mathematics, Kongju National University, Gongju-si, Republic of Korea; ^3^Graduate School of Technology and Innovation Management, Hanyang University, Seoul, Republic of Korea; ^4^Department of Neurology, College of Medicine, Hanyang University, Seoul, Republic of Korea; ^5^Department of Brain and Cognitive Engineering, Korea University, Seoul, Republic of Korea

**Keywords:** mild cognitive impairment, EEG, spectral power, complexity, functional connectivity, graph analysis

## Abstract

Alzheimer's disease (AD) causes a rapid deterioration in cognitive and physical functions, including problem-solving, memory, language, and daily activities. Mild cognitive impairment (MCI) is considered a risk factor for AD, and early diagnosis and treatment of MCI may help slow the progression of AD. Electroencephalography (EEG) analysis has become an increasingly popular tool for developing biomarkers for MCI and AD diagnosis. Compared with healthy elderly, patients with AD showed very clear differences in EEG patterns, but it is inconclusive for MCI. This study aimed to investigate the resting-state EEG features of individuals with MCI (*n* = 12) and cognitively healthy controls (HC) (*n* = 13) with their eyes closed. EEG data were analyzed using spectral power, complexity, functional connectivity, and graph analysis. The results revealed no significant difference in EEG spectral power between the HC and MCI groups. However, we observed significant changes in brain complexity and networks in individuals with MCI compared with HC. Patients with MCI exhibited lower complexity in the middle temporal lobe, lower global efficiency in theta and alpha bands, higher local efficiency in the beta band, lower nodal efficiency in the frontal theta band, and less small-world network topology compared to the HC group. These observed differences may be related to underlying neuropathological alterations associated with MCI progression. The findings highlight the potential of network analysis as a promising tool for the diagnosis of MCI.

## 1. Introduction

Alzheimer's disease (AD) is the most common cause of dementia and is a progressive neurodegenerative disorder (1–3). Currently, over 47 million people worldwide are affected by dementia, this number is expected to increase to approximately 131.5 million by 2050 (4). AD is considered to be caused by the accumulation of beta-amyloid plaques and neurofibrillary tangles in the brain, leading to neuronal dysfunction, and the rapid deterioration of cognitive and physical functions, such as problem-solving, memory, language, and daily activities (5–7). The disease primarily affects older adults, with its prevalence sharply increasing with age, and poses a significant burden on affected individuals, their families, and healthcare systems (8, 9). The pathophysiological process of AD often initiates many years prior to the emergence of clinical symptoms. However, symptoms often do not manifest in the early stages and gradually worsen over time (10, 11). AD is commonly divided into three stages based on symptoms: mild, moderate, and severe (12). As the disease progresses through these stages, patients experience a gradual loss of cognitive functions with synaptic loss and neuronal dysfunction leading to irreversible brain damage in the severe stage. Although a definitive cure for AD is yet to be found, several treatments can slow its progression in the early stages (13). Therefore, early detection of AD is of paramount importance, as it enables timely intervention and management, potentially improving patients' quality of life (14, 15). Mild cognitive impairment (MCI) is a condition characterized by a noticeable decline in cognitive abilities that goes beyond typical age-related changes but does not meet the criteria for dementia owing to its less severe nature (7, 16, 17). Patients with MCI often have an increased risk of developing AD, and thus detecting MCI is crucial to help delay or even prevent its progression to AD (17–19). To achieve this, it is essential to identify reliable biomarkers that can accurately characterize the early stages of AD or MCI compared to normal aging.

Magnetic resonance imaging (MRI) and positron emission tomography are widely used imaging techniques for diagnosing MCI and AD. These methods provide detailed information about the brain's anatomical and network features; however, their usage is restricted owing to the high cost of facilities and the need for specialized expertise. Therefore, electroencephalography (EEG) has increasingly been used to identify biomarkers for MCI diagnosis. EEG is a non-invasive and cost-effective technique that measures postsynaptic potentials of cortical neurons firing synchronously in the brain (20–23). Its relatively safe and quick application makes it particularly suitable for conducting repeated measurements in high-risk older individuals. Several studies have validated the feasibility and reliability of resting-state EEG (rsEEG) in identifying cognitive impairments caused by AD or MCI (24). Various analytical methods, such as spectral properties, synchronization patterns, and network structures have been utilized to uncover EEG biomarkers associated with MCI.

EEG spectral power captures the amplitude of oscillations in each frequency band and can be used as a potential biomarker to differentiate AD from normal aging (25–28). In spectral analysis, it has been predominantly observed that the dementia stages of AD are associated with slowing oscillations caused by increasing low-frequency and decreasing high-frequency band power (24). However, other studies found no or minimal differences between the MCI and healthy control (HC) groups (29–31), suggesting that the neurophysiological changes underlying MCI may not always be apparent in spectral power owing to the complex and heterogeneous conditions of MCI. Therefore, it is important to combine multiple biomarkers such as complexity measures, functional connectivity, and graph-based network analyses, to enhance MCI detection. Entropy analysis is a method used to quantify the complexity of the EEG signals (32–34). Multiscale entropy extends the entropy technique to multiple time scales when the time scale of relevance in a time series, such as EEG, is unknown. It has widely been used to identify AD and MCI patterns and has shown that complexity decreases as AD progresses (35, 36). However, power spectral density (PSD) and entropy-based complexity analyses are limited to assessing a single EEG channel and do not reflect the relationships between channels.

The MCI group has exhibited both structural and functional alterations, indicating that disruptions in the brain network may begin during the MCI stage (37, 38). Consequently, examining the features of brain networks in MCI could be essential for the early diagnosis of AD. Various approaches have been employed to study brain network changes in MCI and AD compared to those in normal aging. Previous studies have shown that patients with AD exhibit altered functional connections between brain regions during the resting state compared to HCs (39–41). Several studies have investigated functional connectivity changes associated with phase synchronization between individuals with MCI and HCs, highlighting abnormal connectivity between brain regions as a key characteristic for identifying MCI conditions (42–46). For instance, the phase lag index (PLI) of EEG functional connectivity in the MCI group showed a considerable decline in synchronization in the delta and theta bands between the frontal and temporal regions (44), while another study reported reduced PLI synchronization in the alpha and beta bands (45). Recently, the weighted PLI (wPLI), which is insensitive to the effects of volume conduction, was applied to functional connectivity analysis but failed to detect significant variations in the MCI group (47). Although EEG functional connectivity has been actively investigated in patients with MCI and AD, there is a lack of consensus. Therefore, the effectiveness of wPLI as an insensitive method for volume conduction of EEG in detecting functional abnormalities in MCI during the resting state remains to be explored.

Graph theory, along with functional connectivity, offers a powerful framework for a better understanding of the structure of brain networks through mathematical models. This enables the analysis of brain network topology, where nodes represent brain regions and links indicate the interactions between these regions (48, 49). The graph model can provide information on the brain's network efficiency, centrality, or small-world properties, thereby enabling the detection of changes in the brain network associated with MCI conditions. Previous studies have shown that AD is correlated with the loss of a small-world network, which reflects the balance between local segregation and global integration within a network (45, 50–53). However, findings on network topology in patients with MCI are inconclusive. Some studies found no significant changes in the MCI network topology compared with the healthy group, while others reported decreased or increased small-worldness (54–56). These inconsistencies may arise from methodological issues, such as thresholding, or variations in connectivity measures and epoch length, which makes comparing networks of different sizes and edge densities challenging and leads to contradictory outcomes (57).

**Figure 1:**
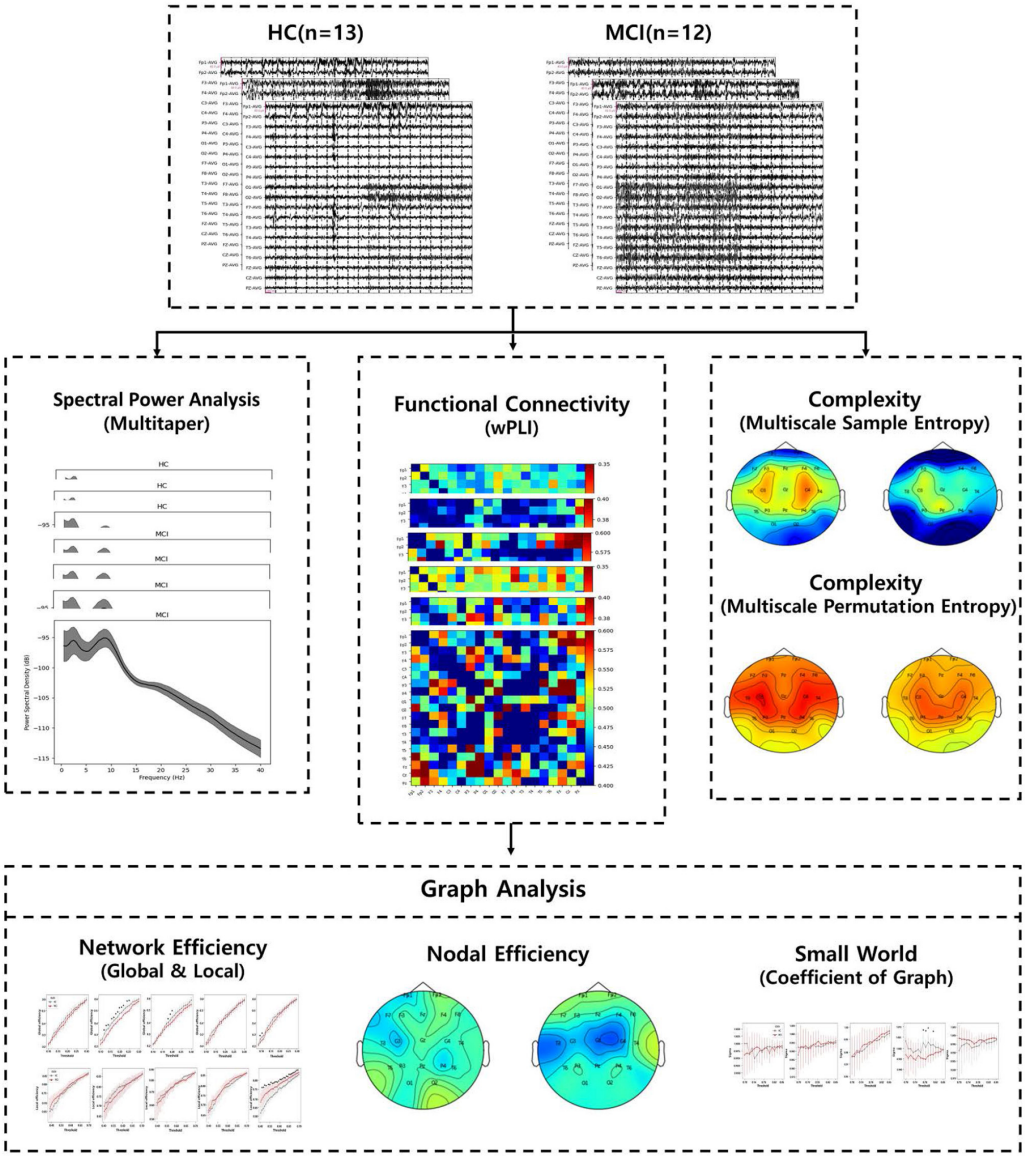
An overview of the entire EEG analytic process.

This study aimed to compare the effectiveness of MCI diagnosis by investigating differences in PSD, complexity, functional connectivity, and graph analysis between individuals with MCI and HCs. An overview of the EEG analysis process is shown in Figure 1. Specifically, wPLI, which is invulnerable to volume conduction, was used across all channels of interest for EEG signals in different bands. The wPLI-based connectivity was investigated for intra- and inter-brain regions: frontal, temporal-parietal, and occipital. Subsequently, brain graph metrics were established using conventional graph measures, considering different thresholds. It is anticipated that conducting graph analysis with variable thresholding will provide further insight into the discriminative features of MCI under resting conditions. These findings demonstrate the potential use of graph theory analysis in the early diagnosis of AD.

## 2. Materials and methods

### 2.1. Participants

This study recruited 25 individuals, from the outpatient memory clinic of the Department of Neurology at Hanyang University Hospital in Seoul and Guri, Korea. Twelve patients were diagnosed with MCI by two neurologists using Petersen's criteria (58). The exclusion criteria included psychiatric disorders that could affect cognition, such as major depressive disorder, a history of alcohol or substance abuse, a history of head trauma, and physical illness that could affect cognitive function. We also excluded patients with structural brain lesions detected by MRI. All participants underwent the Clinical Dementia Rating (CDR) and the Korean Mini-Mental State Examination (K-MMSE). K-MMSE was licensed for use and CDR Sum of Boxes (CDR-SB) scores were obtained for comparisons. We provide a summary of participants' demographic data in Table 1. As shown in Table 1, there was a significant difference in K-MMSE scores between the two groups, while there were no significant differences in terms of sex, education, age, and CDR-SB. Although our data revealed differences in K-MMSE scores between the MCI and HC groups, the scores of the two groups are within the normal range. Furthermore, the K-MMSE is insufficient to differentiate between MCI and normal aging groups accurately due to its limitations in detecting subtle cognitive impairments (59). Informed consent was obtained from each participant according to the recommendations of the Helsinki Declaration. The Institutional Review Board (IRB) of Hanyang University Hospital approved this study (2021-08-020/2021-10-022).

**Table 1:** Demographic and neuropsychological data of healthy controls (HCs) and individuals with mild cognitive impairment (MCI).

**Parameter**	**HC (*n* = 13)**	**MCI (*n* = 12)**	***p*-value**
Age (y)	73 (71–77)	72.5 (65/78)	0.70
Sex (male/female)	8/5 (62%/38%)	5/7 (42%/58%)	0.32
Education (y)	16 (9–16)	12 (3.75/15.25)	0.18
K-MMSE	28.46 (0.97)	26.33 (2.84)	0.02
CDR-SB	0.5 (0.5–1)	1 (0.5/1.5)	0.21

Independent Student's *t*-test for normally distributed data [mean (SD)]; Wilcoxon rank-sum test for non-normally distributed data [median (IQR)]; χ^2^ test for categorical data (%).

IQR, interquartile range; SD, standard deviation.

### 2.2. EEG recording

All participants underwent routine EEG examinations using a 32-channel EEG recording system (COMET-Plus, Grass Technologies Inc., West Warwick, RI, USA). The EEG recordings were performed during daytime hours (9 AM–5 PM) on weekdays for approximately 30 min. The participants were examined in a supine position with eyes closed in a sound-attenuated room. We used 19 electrodes placed according to the international 10–20 system (Fp1, Fp2, F3, F4, F7, F8, C3, C4, T3, T4, T5, T6, P3, P4, O1, O2, Cz, Fz, and Pz) with ear references (A1 and A2). The contact impedance of each electrode was maintained below 10 kΩ. rsEEG data were recorded at a sampling frequency of 200 Hz and filtered with a lowpass filter of 70 Hz.

We applied a bandpass filter from 0.1 to 50 Hz to the rsEEG data. The continuously filtered data from each channel were then divided into 2-s epochs. We visually examined the EEG data and manually rejected epochs with considerable artifacts. For further analysis, we selected 50 artifact-free segments from each participant's channel for a total recording period of 100 s per channel. Estimating the spectral properties from multiple 2-s epochs and subsequently taking the average can improve the frequency resolution of the spectrum while mitigating the effects of noise (60). EEG data preprocessing was conducted using MNE-Python (version 1.4.2) (61).

### 2.3. Spectral power analysis

PSD is a signal processing tool to analyze time-series data, such as EEG signals. It quantifies the power or energy present in a signal at various frequencies. This is useful for understanding the dominant frequencies of a signal, which can reflect the underlying physiological or physical processes. In this study, we calculated the PSD using the multitaper method. PSD was divided into five frequency bands: delta (0.1–4 Hz), theta (4–8 Hz), alpha (8–12 Hz), beta (12–30 Hz), and gamma (30–45 Hz). To identify the topographic distribution of specific bands, we first obtained the mean power spectra of the group-averaged spectrograms across the entire 100-s epoch for each electrode. Subsequently, we averaged the mean power spectra for each EEG frequency band. Topographical power distributions were computed using the topomap function in MNE-Python by performing 2D interpolation of the electrode montage.

### 2.4. Complexity

#### 2.4.1. Multiscale entropy

To quantify the degree of irregularity or complexity in the EEG time-series data across multiple time scales, we employed the widely used multiscale entropy method (62). This involves computing different types of entropy measures on increasingly coarse-grained versions of the original signal. To obtain multiple time scales, the original EEG time-series data [*x*_1_, *x*_2_, …, *x*_*N*_] were coarse-grained using a scale factor τ. Coarse graining is a process where the data is averaged over non-overlapping windows of a certain length determined by the scale factor τ. Each element of the coarse-grained time series *y*_*j*_ was obtained using the following equation:


(1) $$\begin{array}{l} y_{j}=\frac{1}{\tau }\overset{j_{\tau }}{\underset{i=\left(j-1\right)\tau +1}{\sum }}x_{i}\left(1\leq j\leq \frac{N}{\tau }\right). \end{array}$$

Subsequently, we calculated sample and permutation entropies for each time series. The entropies can assess the complexity of EEG signals at different time scales, providing a more comprehensive view of the underlying brain activities. We utilized the NeuroKit2 Python package to compute the multiscale entropies of EEG data (63).

#### 2.4.2. Sample entropy

Sample entropy (SE) is an advanced version of approximate entropy that provides a more robust measure for computing the predictability or regularity of a nonlinear time series (64). To calculate the SE for *y*_*j*_, we derived a sequence of vectors of length *m*, *v*_*m*_(*n*) = [*y*_*n*_, *y*_*n*+1_, ..., *y*_(*n*+*m*−1)_]. We then defined SE as the negative natural logarithm of the conditional probability that two similar sequences of consecutive data points within a time series of length *m* will remain similar at the next point within a specified tolerance level *r*. It is important to note that self-matching at the previous point was excluded. For a fixed embedding dimension *m*>0, fixed time lag, *L* = 1 and tolerance 0 <*r* < 1, the SE for the time series was defined as:


(2) $$\begin{array}{l} SE\left(r,m,N\right)=-log\frac{C_{m+1}\left(r\right)}{C_{m}\left(r\right)} \end{array}$$

*C*_*m*_(*r*) represents an estimate of the probability of two sequences matching for *m* data points, and *C*_*m*+1_(*r*) corresponds to an estimate of the probability of a sequence matching for *m*+1 data points. These probabilities, *C*_*m*_(*r*) and *C*_*m*+1_(*r*), are determined using a relative frequency-based methodology from the provided data. We used a tolerance level of *r* = 0.25 for the maximum distance and *m* = 1 for the length of data sequences denoted by *C*_*m*_. This was used to avoid exponentially increasing the computational cost and improve the efficiency of SE calculation.

#### 2.4.3. Permutation entropy

Permutation entropy (PE) is a measure of the disorder in a time series and is computed by counting the number of unique permutations of the time series within a sliding window of length *m* (65). The PE quantifies the complexity or irregularity of the time series, with higher values indicating greater disorder. To calculate PE of a coarse-grained time series, represented by *y*_*j*_, we constructed a series of vectors of length *m* denoted by *v*_*m*_(*n*) = [*y*_*n*_, *y*_*n*+1_, …, *y*_(*n*+*m*−1)_]. The vectors *v*_*m*_(*n*) were then sorted in ascending order to obtain *m*! possible order patterns, referred to as motifs. The frequency of each motif denoted as *f*(π) and the relative frequency *p*(π) = *f*(π)/(*N*−*m*+1) were computed, where *N* is the total number of samples and τ is the length of the time series.

For a fixed embedding dimension *m*>2 and fixed time-lag *L* = 1, the PE for the time series was defined as:


(3) $$\begin{array}{l} H\left(m\right)=-\overset{m!}{\underset{i=1}{\sum }}p_{i}\left(\pi \right)\log_{2}\left(\pi \right) \end{array}$$

The maximum value of *H*(*m*) is log_2_(*m*!), indicating that all the motifs have an equal probability. The smallest value of *H*(*m*) is zero, suggesting a highly regular time series that repeats with only a few basic motifs. In this study, we used an embedding dimension *m* = 3.

To analyze the complexity, we used two entropy methods, SE and PE, based on multiscale entropy. By selecting scale factors, we captured various levels of complexity and explored the differences in complexity patterns between the HC and MCI groups. Specifically, we divided the data into low scales (1-15) and high scales (16-30) according to the scale factor.

### 2.5. Functional connectivity

To compute functional connectivity, we employed wPLI, an advancement stemming from the PLI (66). The advantage of wPLI lies in the weighting of each phase difference based on the magnitude of the lag. Consequently, phase mismatches near zero have only a minimal influence on wPLI calculations. Therefore, wPLI is highly sensitive for accurately detecting phase interactions of spatially close signals and is more robust to volume conduction compared to PLI, coherence, and virtual coherence. This method reduces the probability of detecting false positive connections and enhances the sensitivity in identifying phase synchronization when faced with volumetric conductive noise sources with near-zero phase delays. The phase lead and lag between two interacting time series were estimated using wPLI as follows:


(4) $$wPLI_{xy}=\frac{n^{-1}\sum_{t=1}^{n}|imag(S_{xyt})|sgn(imag(S_{xyt}))}{n^{-1}\sum_{t=1}^{n}|imag(S_{xyt})|}$$

where *sgn* denotes the sign function and *S*_*xyt*_ represents the cross-spectrum of time series *x* and *y* at time *t*. Only the imaginary part of the cross-spectrum is returned by the *imag* function. The cross-spectrum is weighted by wPLI according to the magnitude of the imaginary components to reduce the effect of small noise on the “real” cross-spectrum signal around the real axis.

In this study, to calculate wPLI, we used five frequency bands: delta (0.1-4 Hz), theta (4-8 Hz), alpha (8-12 Hz), beta (12-30 Hz), and gamma (30-45 Hz). For each frequency band, we calculated the wPLI values for all pairwise combinations of the 19 pre-obtained EEG channels. This yielded a square 19 × 19 weighted adjacency matrix containing the wPLI values for all the channel pairs. Subsequently, we calculated the mean of all pairwise wPLI values to generate a wPLI value representing whole-brain synchronization for each epoch. In addition, we divided the brain into frontal (Fp1, Fp2, F3, F4, F7, F8, and Fz), central (C3, C4, T3, T4, and Cz), and parieto-occipital (P3, P4, O1, O2, T5, T6, and Pz) regions to facilitate comparison of the average wPLI values associated with the connections between these brain regions. All functional connectivity analyses were conducted using MNE-Python functions (61).

### 2.6. Graph analysis

#### 2.6.1. Graph theory approach

A graph, which is a mathematical structure composed of nodes and edges, is used to represent a brain network (67). The nodes represent brain regions, and the edges represent the connections between these regions. We used an undirected binary graph representation, designating all connections as either 1 or 0, without considering the directionality of the edges. A graphical representation of the functional brain network was constructed based on the wPLI, which measures functional connectivity. We chose the wPLI because of its robustness to volume conduction. Considering the phase lag between neural oscillations, the wPLI provides a reliable estimation of functional connectivity that is less affected by indirect connections. Moreover, when the number of nodes is small, the wPLI has been shown to outperform the other connectivity measures, making it a good choice for our study (66). The sparsity threshold (S) plays an important role in analyzing functional connectivity between brain regions. It is determined by dividing the total number of edges in a graph by the maximum possible number of edges that is given by *n*(*n*−1)/2 for a graph with *n* nodes. In this case, the maximum possible number of edges was $\frac{19\times 18}{2}=171$. The sparsity threshold determines the number of edges in the graph. For example, if *S* = 0.1, only 10% of all edges are retained, whereas the rest are considered unconnected. This procedure enabled the identification of significant brain connections.

As there is no standardized method for selecting a single sparsity threshold, we repeatedly changed the threshold of the inter-region correlation matrix by 1% over a sparsity range (10% ≤ *S* ≤ 80%). To achieve this, we selected a sparsity threshold and transformed our data into a binary matrix by selecting the top *N* strongest connections, where *N* = threshold × 171. By focusing on strong connections, we were able to analyze meaningful connections within the brain networks (68). Following this thresholding procedure, we computed both global and local network efficiency, nodal efficiency, and small-world properties using the NetworkX Python package (69).

#### 2.6.2. Network efficiency

To investigate the integrated information, we calculated the global efficiency. Network integration is the ability to combine information from different brain regions and transmit it through the network. We first obtained the connectivity matrix for each individual, and then applied a sparsity threshold to obtain a corresponding binary adjacency matrix. From each adjacency matrix, we constructed a graph and computed both global and local efficiencies. Global efficiency measures how efficiently the information can be transferred across the entire graph by measuring the average inverse of the shortest path length from one node to all the other nodes in the graph (70). Although global efficiency and characteristic path length are linearly correlated (71), global efficiency has the advantage that it considers all the shortest paths equally and not the few longest shortest paths, which can provide a more balanced measure of network integration (49). Specifically, given a graph *G*, the global efficiency, *E*_*glob*_(*G*), is


(5) $$\begin{array}{l} E_{glob}\left(G\right)=\frac{1}{N\left(N-1\right)}\underset{i\neq j\in G}{\sum }\frac{1}{d\left(i,j\right)} \end{array}$$

where *N* is the number of nodes in the graph, and *d*(*i, j*) is the shortest path length between nodes *i* and *j*. In addition, *d*(*i, j*) of unconnected nodes is set to infinity to force the inverse to zero when calculating global efficiency or characteristic path length.

In contrast, local efficiency refers to the efficiency of information flow over subgraphs of a graph. It calculates the efficiency of the local subgraph surrounding each node in a graph and provides an average measure of efficiency across all nodes in the graph (70). The local efficiency of a graph *G*, *E*_*loc*_(*G*), is defined as:


(6) $$\begin{array}{l} E_{loc}\left(G\right)=\frac{1}{N}\underset{i\in G}{\sum }E_{glob}\left(G_{i}\right) \end{array}$$

where *G*_*i*_ denotes a subgraph, and *i* denotes nodes in the graph.

#### 2.6.3. Nodal efficiency

Nodal efficiency is a measure closely related to global efficiency and is computed for each individual node in the graph. It reflects the efficiency of communication between a given node and its immediate neighbors. Nodal efficiency can be defined mathematically as follows: Let *G* be a graph with *N* nodes, and let *G*_*i*_ be a subgraph consisting of the neighbors of node *i* and its edges. The nodal efficiency *E*_*nodal*_ of node *i* is then given by:


(7) $$\begin{array}{l} E_{nodal}\left(i\right)=\frac{1}{N-1}\underset{j\neq i}{\sum }E_{glob}\left(G_{i}\right) \end{array}$$

where *E*_*glob*_(*G*_*i*_) is the global efficiency of subgraph *G*_*i*_, and the sum is taken over all nodes in the graph except for node *i*. Intuitively, nodal efficiency captures how efficiently information can be transmitted from a given node to the rest of the network through its immediate neighbors. A node with high nodal efficiency can communicate effectively with other nodes in the network, whereas a node with low nodal efficiency may be relatively isolated from the other nodes of the network (72, 73).

#### 2.6.4. Small world

A small-world network is a mathematical graph in which most nodes are not neighbors, but are indirectly interconnected through shared neighbor nodes. This means that while a specific node may be directly linked to many others, its neighbors are connected with small characteristic path lengths (74). Consequently, small-world networks exhibit an intriguing blend of the characteristics observed in regular lattices and random graphs. Regular lattices are highly clustered, meaning that nodes are tightly knit into groups where each member shares multiple connections with others in the group. However, random graphs have small characteristic path lengths, indicating relatively few steps or hops between any two nodes. Essentially, the small-world is a hybrid structure. To quantify small-worldness, we typically use the network's average clustering coefficient (C) and average characteristic path length (L). The average clustering coefficient quantifies how closely nodes in a network tend to cluster together. It is defined as the ratio of the number of connections between a node's neighbors to the total number of possible connections between them. More formally, the clustering coefficient of node *i* in graph *G* is defined as:


(8) $$\begin{array}{l} C_{i}=\frac{2e_{i}}{k_{i}\left(k_{i}-1\right)} \end{array}$$

where *e*_*i*_ represents the number of edges between neighbors of node *i*, and *k*_*i*_ is the number of neighbors of node *i*. The factor of 2 in the numerator accounts for the fact that each edge contributes to the clustering coefficient of both of the nodes it connects. The average characteristic path length counts the average number of steps needed to connect any two nodes in a network, considering the shortest possible paths, which can be a measure of the average distance between all possible pairs of nodes. The average clustering coefficient *C* of a network is the average of the clustering coefficient *C*_*i*_ for all nodes *i* in the network:


(9) $$\begin{array}{l} C=\frac{1}{N}\underset{i\in G}{\sum }C_{i} \end{array}$$

where *N* is the number of nodes in the network, and the sum represents the sum of all nodes in the network. The characteristic path length is the average shortest path length between all pairs of nodes in the network:


(10) $$\begin{array}{l} L=\underset{i\neq j\in G}{\sum }\frac{d\left(i,j\right)}{N\times \left(N-1\right)} \end{array}$$

where *N* is the number of nodes in the network and *d*(*i, j*) is the shortest path length between nodes *i* and *j*. In addition, *d*(*i, j*) of unconnected nodes is set to infinity to force the inverse to zero in the calculation of global efficiency or characteristic path length. and ∑ represents the sum of all pairs of nodes. To measure the small-worldness of the actual network we computed the small-world coefficient σ using the formula:


(11) $$\begin{array}{l} \sigma =\frac{C/C_{r}}{L/L_{r}} \end{array}$$

where *C* and *L* are the average clustering coefficient and the average shortest path length of *G*, respectively. *C*_*r*_ and *L*_*r*_ are the average clustering coefficient and the average shortest path length, respectively, across 100 equivalent random graphs created using MNE-Python library. A graph is commonly classified as a small world if σ>1 (75).

### 2.7. Statistical analysis

We used different statistical tests to evaluate demographic and clinical differences between the two groups, depending on the nature of the data and its distribution. For continuous variables, we first conducted the Shapiro-Wilk test to determine whether each variable followed a normal distribution. If a variable was normally distributed, an independent Student's *t*-test was applied to assess the difference between the two groups; otherwise, the Wilcoxon rank-sum test was used. For categorical variables, the χ^2^ test was implemented. Results for continuous data were represented either as arithmetic means with standard deviations (SD) for normally distributed data or medians with interquartile ranges (IQR) for non-normally distributed data. Categorical data were reported as frequencies (%).

We also performed statistical analyses on multiple EEG measures: PSD, complexity, functional connectivity, network efficiency, nodal efficiency, and small world. We used the Wilcoxon rank-sum test to compare the measures between the two groups and determine if their distributions were significantly different. Specifically, we calculated the rank test statistics for each measure and obtained the corresponding *p*-values. A *p*-value of less than 0.05 was considered statistically significant.

We also adjusted the *p*-values for PSD, complexity, functional connectivity, and nodal efficiency using a false discovery rate (FDR) test to control for multiple comparisons excluding network efficiency and small world. We used the Benjamini-Hochberg method to control the FDR when conducting multiple simultaneous hypothesis tests (76). For the analyses of PSD, complexity, and nodal efficiency, the FDR approach was applied separately within each frequency band across all nineteen electrodes. The procedure involved ranking the *p*-values in ascending order, and then computing the adjusted *p*-value for the *i*th smallest *p*-value, *p*(*i*), using the formula,


(12) $$\begin{array}{l} p_{{}_{\text{FDR}}}\left(i\right)=\min\left\{N/i\times p\left(i\right),p_{{}_{\text{FDR}}}\left(i-1\right)\right\}, \end{array}$$

where *p*__FDR__(0) is defined as 1, and *N* is the number of multiple comparisons (*N* = 19; nineteen electrodes). In the functional connectivity analysis, *p*-values were computed for 171 connections within each frequency band, and then the FDR approach was applied to the set of *p*-values from five frequency bands, separately at each connection. This method helped control the expected proportion of false discoveries (i.e., the erroneous rejection of null hypotheses), thereby reducing the likelihood of false positives and improving the overall validity of our results. We considered an adjusted *p*-value (*p*__FDR__) of 0.05 or lower after *post-hoc* correction to be statistically significant.

Furthermore, we used a nonparametric bootstrapping method to represent the uncertainty of data in complexity, network efficiency, and small world. The bootstrapping procedure involved resampling the dataset 1,000 times with replacement and recalculating the mean from each resample. From this bootstrapped distribution, we constructed 95% confidence intervals, which are represented by the error bars in the plots.

## 3. Results

### 3.1. Power spectral properties

**Figure 2:**
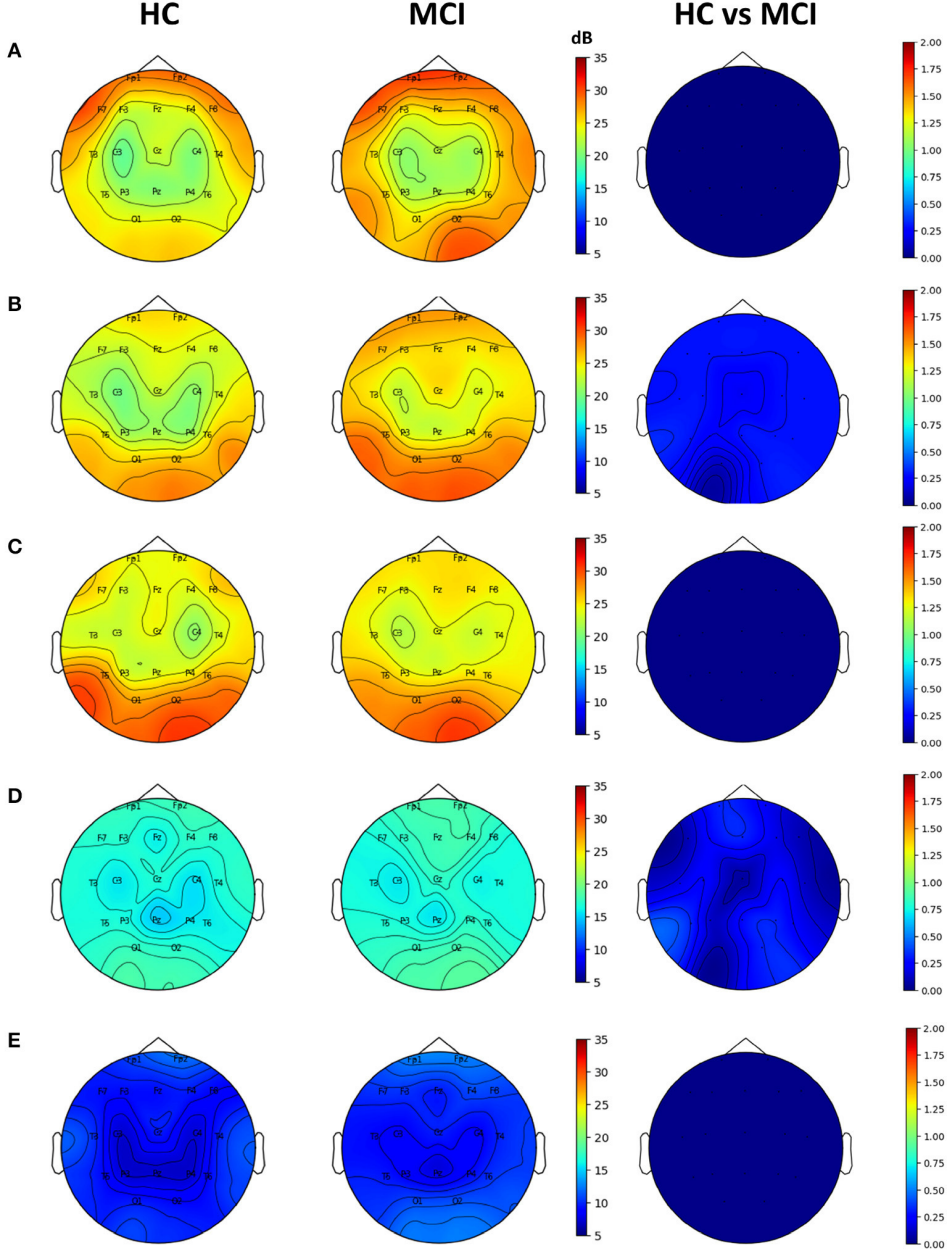
Topographical EEG maps of group-averaged spectral power and statistics on group differences in the **(A)** delta, **(B)** theta, **(C)** alpha, **(D)** beta, and **(E)** gamma bands from top to bottom row. The first and second columns are for the HC and MCI groups, respectively. The color scale is in decibels. The third column demonstrates the statistical differences in spectral power between the HC and MCI groups (*p* < 0.05, FDR-corrected). The color represents negative log *p*-values.

Topographic plots were computed for the delta (0.1–4 Hz), theta (4-8 Hz), alpha (8–12 Hz), beta (12–30 Hz), and gamma (30–45 Hz) frequency bands in the HC and MCI groups. In both groups, delta oscillations were dominant in the frontal area (Fp1 and Fp2) (Figure 2A). Theta and alpha oscillations were prominent in occipital regions (O1 and O2) in two groups (Figures 2B, C). Beta and gamma activities remained negligible across all brain regions (Figures 2D, E). However, no significant differences were found between the HC and MCI groups in any of the frequency bands and channels after FDR correction.

### 3.2. Complexity analysis

**Figure 3:**
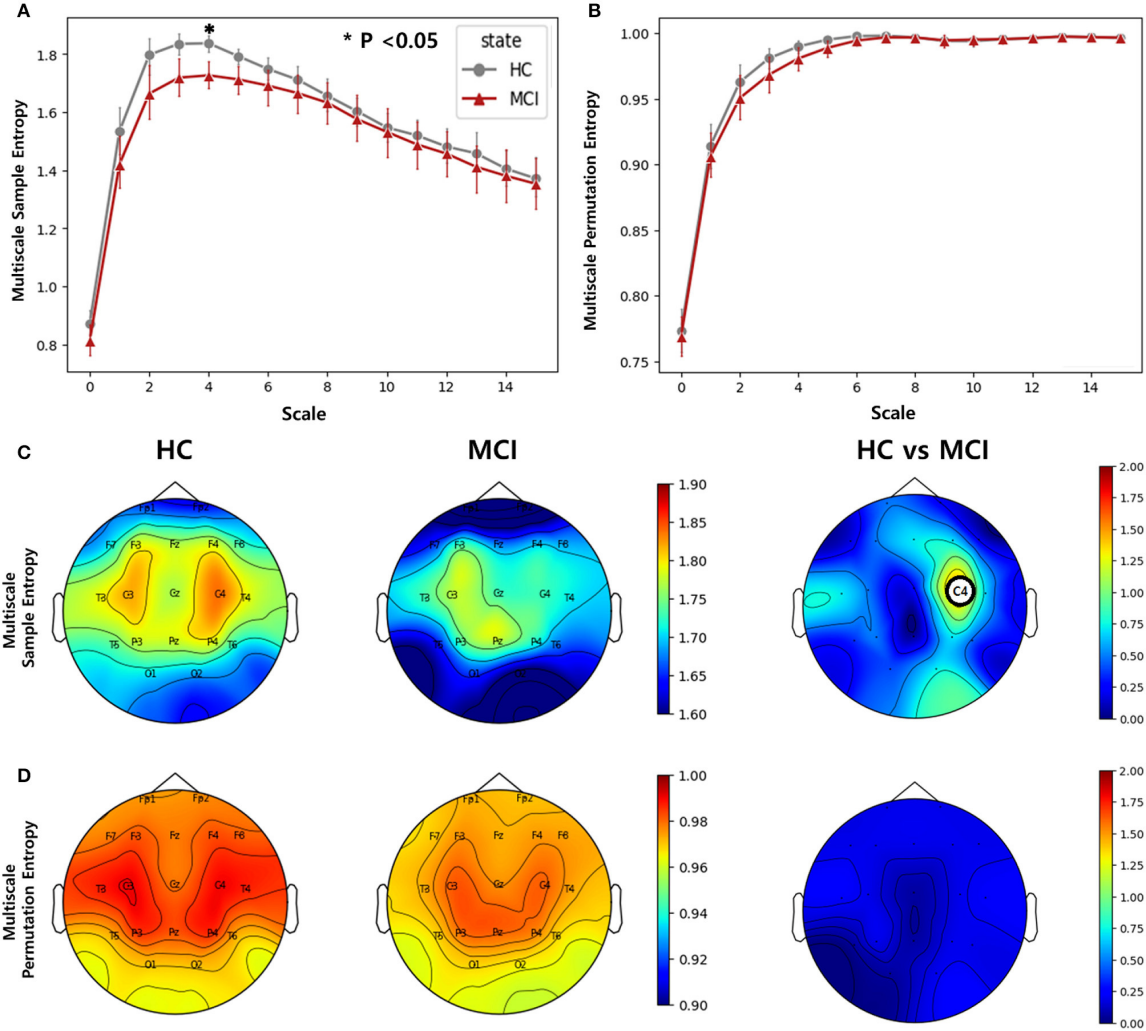
Complexity comparisons between the HC and MCI groups across varying scale factors from 1 to 15 using **(A)** multiscale sample entropy and **(B)** multiscale permutation entropy. Error bars represent 95% confidence intervals. Topographical distributions of group-averaged entropy at scale of 4 and statistics on group difference for **(C)** multiscale sample entropy, and **(D)** multiscale permutation entropy. The first and second columns are for the HC and MCI groups, respectively. The third column demonstrates the statistical difference in entropy between the HC and MCI groups (*p* < 0.05, FDR-corrected). The asterisk and circle on the electrode in the topographical map represent statistical significance. The color represents negative log *p*-values.

We computed multiscale SE and multiscale PE using scale factors ranging from 0 to 30 for all channels. In the entropy analysis, the averaged entropy values over all channels of the MCI and HC groups were different at low-scale factors (0 to 15). The averaged multiscale SE showed a significant difference at the scale factor of 4 between the two groups, while the averaged multiscale PE showed no significant difference (Figures 3A, B). In the topographic plots, the multiscale SE with a scale of 4 showed that the HC group had a higher complexity in the middle temporal lobe (C3 and C4) than in the other channels. However, the MCI group exhibited a reduced complexity in the region. Consequently, in the C4 channel, there was a statistically significant difference between the two groups (*p*=0.0019, *p*__FDR__ = 0.037) (Figure 3C). Regarding the multiscale PE analysis with a scale factor of 4, similar patterns were observed, where the HC group had a higher complexity in the middle temporal lobe than the MCI group, however, no significant difference was found between the HC and MCI groups across all channels (Figure 3D).

### 3.3. Functional connectivity

**Figure 4:**
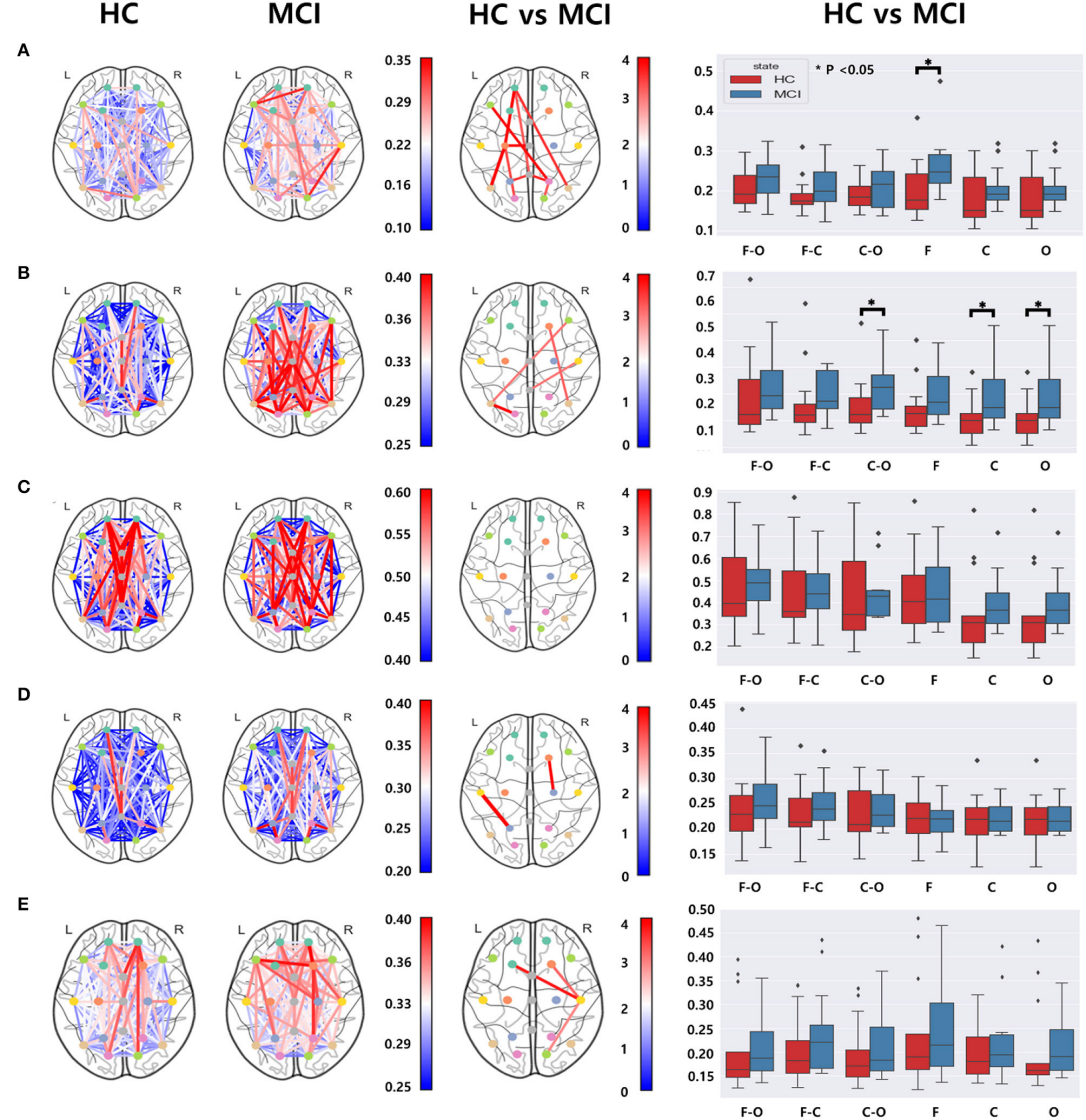
Functional connectivity comparisons between the HC and MCI groups using pairwise wPLI in the **(A)** delta, **(B)** theta, **(C)** alpha, **(D)** beta, and **(E)** gamma bands. The first and second columns represent topographical connections in wPLI for the HC and MCI groups, respectively. The color-coded lines represent wPLI values. The third column demonstrates the topographical changes in wPLI between the HC and MCI groups. The color represents negative log *p*-values. The fourth column shows comparisons of wPLI distributions intra- and inter-regions: F-frontal (Fp1, Fp2, F3, F4, F7, F8, and Fz), C-central (C3, C4, T3, T4, and Cz), and O-parieto-occipital (P3, P4, O1, O2, T5, T6, and Pz) regions. In a box plot, a box is drawn from the first quartile to the third quartile. A horizontal line within the rectangle represents the median of all values. The asterisks represent statistical significance (*p* < 0.05, FDR-corrected).

We utilized the wPLI values computed from 171 different electrode pairs for each frequency band: delta, theta, alpha, beta, and gamma. We compared these wPLI values to examine any discrepancies between the HC and MCI groups. Significant differences were observed in wPLI values between the MCI and HC groups for all frequency bands except the alpha band (Figure 4). With regard to the connectivity of the frontal and centro-temporal lobes, the MCI group exhibited higher wPLI values than the HC group in one pair within the delta and beta frequency bands (Figures 4A, D) and two pairs in the gamma frequency band (Figure 4E). For the theta and alpha frequency bands, the connectivity in the frontal and centro-temporal lobes showed no significant differences between the two groups. However, the connectivity between the frontal and occipital lobes showed significant differences between the two groups, which was noticeable over three pairs in the delta frequency band (Figure 4B) and two pairs in the theta frequency band (Figure 4B). For the connection between the central and occipital lobes, the HC group had lower wPLI values than the MCI group in two pairs in the delta frequency band(Figure 4A) and in one pair within the theta, beta, and gamma frequency bands (Figures 4B, D, E). However, in the alpha frequency band, the differences between the two groups were not statistically significant. Moreover, we analyzed the differences in the average wPLI values across brain regions: frontal (Fp1, Fp2, F3, F4, F7, F8, and Fz), central (C3, C4, Cz T3, and T4), and parieto-occipital (P3, P4, O1, O2, T5, T6, and Pz). All wPLI values were averaged over all intra- and inter-regional pairs. In the delta frequency band, the MCI group demonstrated a significantly higher average wPLI value over the intra-channels in the frontal lobe compared to those in the HC group (Figure 4A). In the theta band, the HC group showed significantly lower values compared to values of the MCI group within the centro-temporal and occipital lobes, as well as between the central and occipital lobes (Figure 4B). However, in the alpha, beta, and gamma bands, wPLI values did not show any significant differences (Figures 4C–E).

### 3.4. Graph analysis

#### 3.4.1. Network efficiency

**Figure 5:**
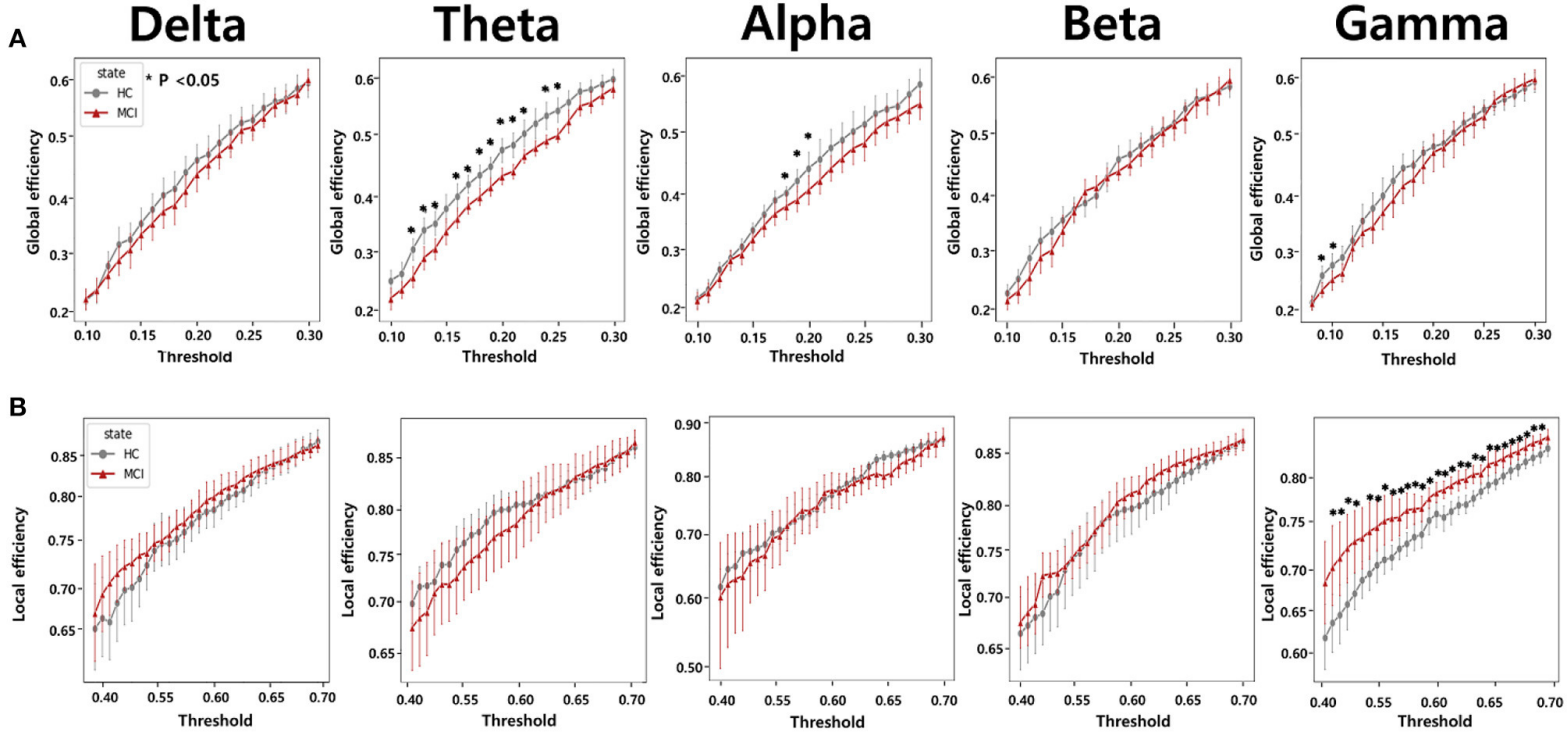
Network efficiency comparisons according to varying thresholds from 0.1 to 0.3 for **(A)** global efficiency and from 0.4 to 0.7 for **(B)** local efficiency between the HC and MCI groups. The five columns represent network efficiencies in the delta, theta, alpha, beta, and gamma bands, respectively. The asterisks represent statistical significance (*p* < 0.05, FDR-corrected). Error bars represent 95% confidence intervals.

To calculate the global efficiency, we performed the analysis by setting the sparsity threshold from 10% to 70% (Figure 5A). Although no statistically significant differences were observed between the beta and delta bands, the HC group exhibited significantly higher global efficiency compared to the MCI group in the alpha, theta, and gamma bands. In the theta band, we observed a significant difference in the global efficiency between the two groups when the sparsity threshold was varied from 12% to 25%. In the alpha band, higher global efficiency values were observed in the HC group than in the MCI group when the sparsity threshold was in the range of 18% to 20%. Moreover, a notable difference was observed in the gamma band, with sparsity thresholds ranging from 10% to 11%.

We also analyzed local efficiency using sparsity thresholds ranging from 10% to 70% (Figure 5B). We observed a clear difference in the gamma band between the HC and MCI groups when the sparsity threshold ranged from 42% to 70%. In the remaining frequency bands, there were no statistically significant differences in local efficiency between the HC and MCI groups. In contrast to the results obtained for global efficiency, the MCI group tended to have a higher local efficiency than the HC group.

#### 3.4.2. Nodal efficiency

**Figure 6:**
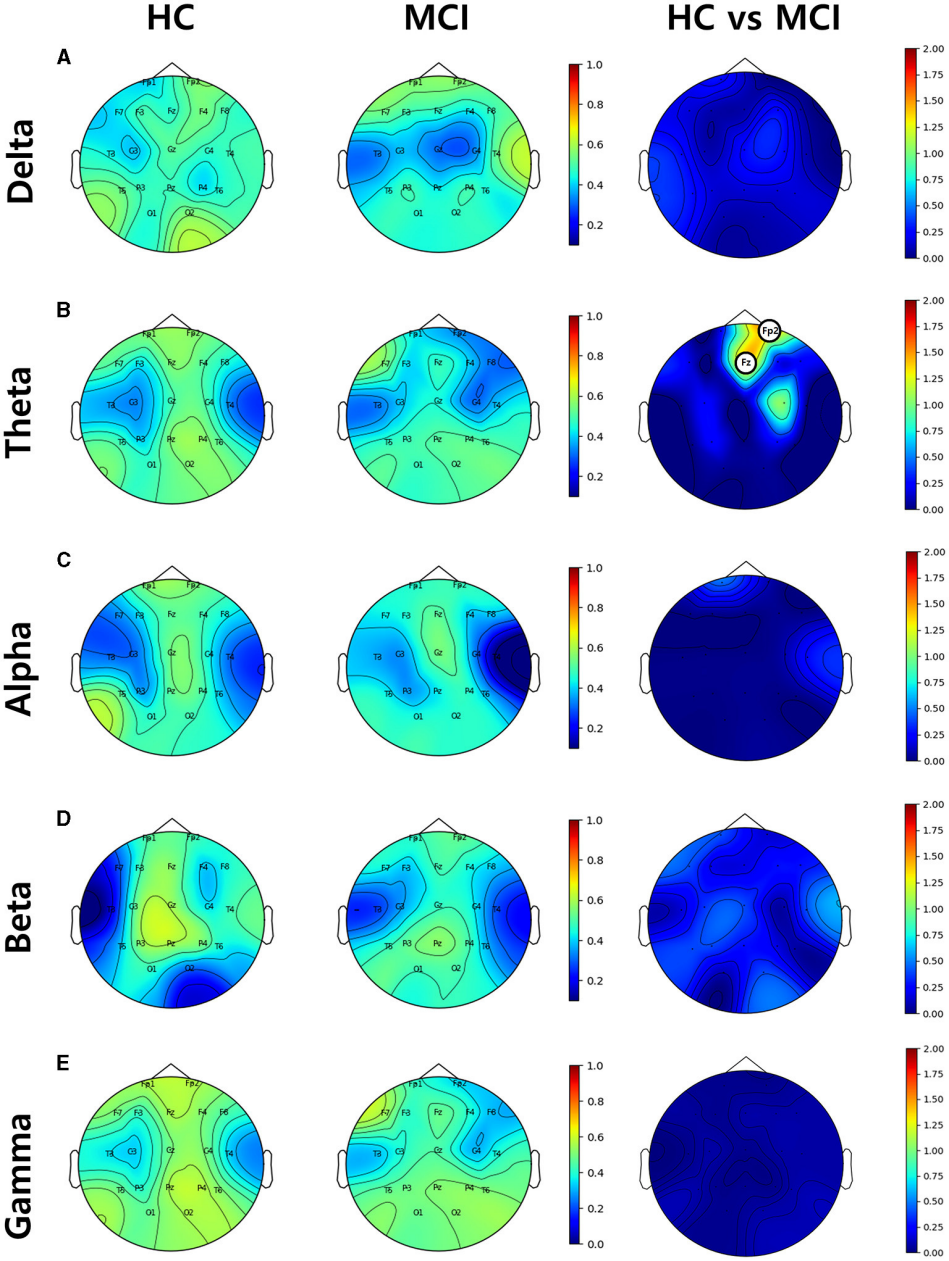
Topographical distributions of group-averaged nodal efficiency and statistics on group difference in the **(A)** delta, **(B)** theta, **(C)** alpha, **(D)** beta, and **(E)** gamma bands from top to bottom row. The first and second columns are nodal efficiencies for the HC and MCI groups, respectively. The third column demonstrates the statistical differences in nodal efficiency between the HC and MCI groups and the circled channels represent statistical significance (*p* < 0.05, FDR-corrected). The circle on the electrode represents statistical significance. The color represents negative log *p*-values.

Nodal efficiency was analyzed by setting the sparsity threshold from 10% to 70%. Significant differences were observed between the HC and MCI groups in the theta band. Specifically, at sparsity thresholds of 13% to 16% and 20% to 22%, the HC group showed higher nodal efficiency in the frontal regions Fz and Fp2, than the MCI group. The 20% threshold had the most significant difference between the groups (Figure 6). The analysis showed that there was a difference between the HC and MCI groups only in the theta band, and no significant differences were found in the rest of the frequency domains.

Specifically, at sparsity thresholds of 13% to 16% and 20% to 22%, the HC group showed higher node efficiency in the frontal region, Fz (*p* = 0.004, *p*__FDR__ = 0.039) and Fp2 (*p* = 0.002, *p*__FDR__ = 0.039), than the MCI group, with the threshold of 20% being the most statistically significant.

#### 3.4.3. Small world

**Figure 7:**
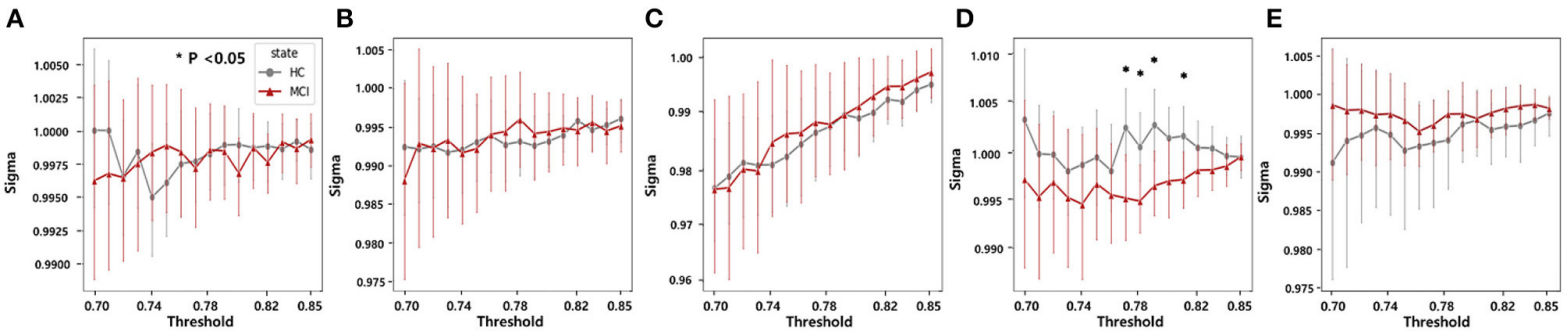
Statistical comparisons of the small-world property (*sigma*) between the HC and MCI groups in a sparsity threshold range of 0.7–0.85 in the **(A)** delta, **(B)** theta, **(C)** alpha, **(D)** beta, and **(E)** gamma bands. The asterisks represent statistical significance (*p* < 0.05, FDR-corrected). Error bars represent 95% confidence intervals.

In each frequency band, we examined small-world networks using high sparsity thresholds ranging between 75% and 85%. These thresholds were selected to ensure that the graph was not excessively disconnected, allowing for the effective examination of small-world networks. The difference between the MCI and HC groups was statistically significant for small-world networks within a sparsity threshold of 76% to 80% in the beta band. Consequently, the MCI group exhibited lower small-worldness in the beta band (Figure 7). However, the rest of the frequency bands did not show any distinct characteristics.

## 4. Discussion

This study aimed to compare EEG features between cognitively healthy controls and age-matched individuals with MCI at rest with their eyes closed. The two groups were compared in terms of spectral power, complexity, functional connectivity, and graph theory-based measures. Our findings indicated that the power spectrum analysis did not yield any significant differences between the HC and MCI groups. However, when analyzing the multiscale SE, we observed that the MCI group exhibited lower complexity. In addition, the MCI group demonstrated higher wPLI values than the HC group. Further examination using graph theory analysis revealed that the MCI group predominantly displayed lower global efficiency in the theta band and higher local efficiency in the gamma band relative to the HC group. Moreover, nodal efficiency was reduced in the frontal region of the MCI group. Finally, in the small-world analysis, the MCI group exhibited a lower small-world coefficient when compared to the HC group. One of the main challenges in functional connectivity analysis is the lack of a definite method for setting an optimal threshold value. The thresholding procedure aims to remove weak edges to eliminate spurious connections; however, the network structure can vary significantly based on the chosen threshold. Consequently, exploring a wide range of threshold values, as in our study, is crucial for ensuring a comprehensive analysis.

In the power spectral analysis, no significant differences were found between the HC and MCI groups in any frequency band (delta, theta, alpha, beta, or gamma). This suggests that there were no significant differences in the overall power of the EEG signals between the two groups. These results are consistent with those of the recent studies suggesting that PSD had a limited ability to distinguish between HC and MCI groups (30, 77). However, some prior findings have revealed that MCI can be characterized by lower alpha and beta power as well as stronger delta and theta power (26). This contradiction may be because the neurophysiological changes associated with MCI may not always be apparent in spectral power, suggesting that the power spectral analysis is relatively insensitive to network changes resulting from MCI progression.

The multiscale SE analysis revealed that the MCI group had significantly lower complexity in the middle temporal lobe (C4) compared to the HC group at a scale factor of 4. This result is consistent with previous findings of reduced complexity at lower scales with the progression of AD (35). This finding indicated that the HC group exhibited more organized and efficient neural activity in this region, and it declined due to the progression of MCI. However, the multiscale PE analysis did not reveal a statistically significant difference between the two groups, despite the observed decrease in complexity with AD progression (78). The Multiscale SE may be more sensitive to the changes in complexity by MCI compared with the multiscale PE method that was effective for AD discrimination. We explored the effect of varying scale factors and found a significant difference at a scale factor of 4, indicating that scale factors should be carefully considered in multiscale complexity analysis.

Regarding functional connectivity, there were significant differences in wPLI values between the HC and MCI groups within the delta, theta, beta, and gamma bands. These distinctions are likely attributable to the robustness of the wPLI to volume conduction and its ability to provide a reliable estimation that is less influenced by indirect connections. Specifically, in the delta band, most connections between channels increased because of MCI. This result is similar to those of previous studies reporting increased functional connectivity in patients with MCI than in HCs in the delta frequency range (30, 42). The MCI group showed higher wPLI values between the frontal and centro-temporal lobes in the delta, beta, and gamma bands; between the frontal and occipital lobes in the delta and theta bands, and between the central and occipital lobes in all bands except the alpha band. Moreover, for the averaged wPLI values across intra- and inter-regions, the MCI group had higher wPLI values within the frontal lobe in the delta band, within central and occipital lobes, and between central and occipital lobes in the theta band. In our study, there was no significant difference in alpha connections even though alpha oscillations are associated with cognitive and perceptual processes, including memory and attention. This may reflect a compensatory mechanism in the early stages of cognitive decline owing to disrupted functional connectivity in brain regions, as some neurons lose their functionality, others may increase their connections to compensate for this subtle loss. This process is known as neuroplasticity that the brain can reorganize itself by forming new neural connections and pathways.

From a network efficiency perspective in graph theory, our study revealed that global efficiency was significantly higher in the HC group than in the MCI group in the theta band in the range of thresholds from 0.13 to 0.26. Some previous studies have also shown decreased global efficiency in patients with MCI compared to HC in the theta band (50, 79). Moreover, in the alpha band, we noticed a decline in global efficiency in the MCI group, specifically over the threshold range of 0.19 and 0.21. However, no significant difference in functional connectivity was found between the two groups. In addition, as in previous studies (80), we observed an increase in local efficiency within the MCI group compared with the HC group. In contrast, observed global efficiency was dominant in the gamma band across a threshold range of 0.42 and 0.7. This finding suggests a deterioration in global efficiency accompanied by an increase in local efficiency during the progression from HC to MCI. Furthermore, the nodal efficiency analysis revealed that the prefrontal region (Fz and Fp2) indicated a statistically significant decrease in the theta band for the MCI group compared to the HC group. These results suggest that there might be regional differences in information processing efficiency between the two groups. Future studies should investigate the role of these regions in MCI pathophysiology. We also examined small-world properties in brain networks and found that the MCI group had a lower small-world coefficient (σ) in the beta band compared to the HC group. Lower small-worldness could potentially suggest alterations or disruptions in the balance between local specialization and global integration, which could have implications for information processing in the brain, therefore, the small-world property may be a potential biomarker for MCI detection.

However, this study has several limitations. First, the relatively small sample size of the participants in each group (*n* = 13 for HC and *n* = 12 for MCI) might have limited the statistical power of the results of this study. Low statistical power reduces the chance of detecting a true effect and increases the likelihood of more false-positive results (81). Therefore, the findings may not be generalizable to large populations. Future studies with larger cohorts would be necessary to validate the relationship between EEG features and MCI. Second, we only investigated EEG patterns during the resting state. However, previous research has shown that the EEG characteristics before and after memory tasks can be more discriminative between the HC and MCI groups (31, 82). Therefore, further studies should record EEG activity before and after the tasks for a better understanding of the relationship between EEG patterns and MCI. Finally, this study was cross-sectional, and the participants were assessed only at a single point in time. Longitudinal studies are necessary to provide additional insight into how these EEG features change along with the progression of AD dementia from MCI.

## 5. Conclusion

Our study aimed to identify the changes in network properties such as complexity, functional connectivity, global efficiency, nodal efficiency, and small-world, that could characterize MCI through the analysis of rsEEG data. By employing the wPLI combined with variable thresholding, we were able to shed light on the functional network and graph structure alterations associated with MCI. Our findings suggest that alterations in functional connectivity and graph theory-based measures, particularly in the theta band associated with memory and attention processes, may serve as important clinical biomarkers for detecting and monitoring cognitive decline in individuals with MCI. However, the findings of this study should be interpreted with caution due to the small sample size. Future studies with larger cohorts and longitudinal designs are necessary to confirm these findings and gain a deeper understanding of the relationship between EEG patterns and MCI.

## Data availability statement

The raw data supporting the conclusions of this article will be made available by the authors, without undue reservation.

## Ethics statement

The studies involving humans were approved by Institutional Review Board (IRB) of Hanyang University Hospital (2021-08-020/2021-10-022). The studies were conducted in accordance with the local legislation and institutional requirements. The participants provided their written informed consent to participate in this study.

## Author contributions

S-EK: supervised the data analyses. S-EK, CS, JY, and B-KM: wrote the manuscript. S-EK, CS, and JY: analyzed the data. KS and HR: designed the project and received the funding. JP and HC: obtained institutional review board approval and collected data. S-EK, CS, KS, JP, HC, HR, and B-KM: reviewed the manuscript. All the authors contributed to the manuscript and approved the submitted version.

## References

[1] Raza M Awais M Ellahi W Aslam N Nguyen HX Le-Minh H. Diagnosis and monitoring of Alzheimer's patients using classical and deep learning techniques. Expert Syst Appl. (2019) 136:353–64. 10.1016/j.eswa.2019.06.03835546606

[2] Safi MS Safi SMM. Early detection of Alzheimer's disease from EEG signals using Hjorth parameters. Biomed Signal Process Control. (2021) 65:102338. 10.1016/j.bspc.2020.1023381777528

[3] Gaugler J James B Johnson T Reimer J Solis M Weuve J. Alzheimer's disease facts and figures. Alzheimers Dement. (2022) 18:700–89. 10.1002/alz.12638 35289055

[4] Sosa-Ortiz AL Acosta-Castillo I Prince MJ. Epidemiology of dementias and Alzheimer's disease. Arch Med Res. (2012) 43:600–8. 10.1016/j.arcmed.2012.11.003 23159715

[5] Hardy J Selkoe DJ. The amyloid hypothesis of Alzheimer's disease: progress and problems on the road to therapeutics. Science. (2002) 297:353–6. 10.1126/science.1072994 12130773

[6] Sperling RA Aisen PS Beckett LA Bennett DA Craft S Fagan AM. Toward defining the preclinical stages of Alzheimer's disease: recommendations from the National Institute on Aging-Alzheimer's Association workgroups on diagnostic guidelines for Alzheimer's disease. Alzheimers Dement. (2011) 7:280–92. 10.1016/j.jalz.2011.03.003 21514248 PMC3220946

[7] Jack CR Jr Bennett DA Blennow K Carrillo MC Dunn B Haeberlein SB. NIA-AA research framework: toward a biological definition of Alzheimer's disease. Alzheimers Dement. (2018) 14:535–62. 10.1016/j.jalz.2018.02.018 29653606 PMC5958625

[8] Brookmeyer R Johnson E Ziegler-Graham K Arrighi HM. Forecasting the global burden of Alzheimer's disease. Alzheimers Dement. (2007) 3:186–91. 10.1016/j.jalz.2007.04.381 19595937

[9] Qiu C Kivipelto M von Strauss E. Epidemiology of Alzheimer's disease: occurrence, determinants, and strategies toward intervention. Dialogues Clin Neurosci. (2009) 11:111. 10.31887/DCNS.2009.11.2/cqiu 19585947 PMC3181909

[10] Bateman RJ Xiong C Benzinger TL Fagan AM Goate A Fox NC. Clinical and biomarker changes in dominantly inherited Alzheimer's disease. N Engl J Med. (2012) 367:795–804. 10.1056/NEJMoa1202753 22784036 PMC3474597

[11] Villemagne VL Burnham S Bourgeat P Brown B Ellis KA Salvado O. Amyloid β deposition, neurodegeneration, and cognitive decline in sporadic Alzheimer's disease: a prospective cohort study. Lancet Neurol. (2013) 12:357–67. 10.1016/S1474-4422(13)70044-9 23477989

[12] Petersen RC. Early diagnosis of Alzheimer's disease: is MCI too late? Curr Alzheimer Res. (2009) 6:324–30. 10.2174/156720509788929237 19689230 PMC3098139

[13] Rasmussen J Langerman H. Alzheimer's disease-why we need early diagnosis. Degener Neurol Neuromuscul Dis. (2019) 9:123–130. 10.2147/DNND.S228939 31920420 PMC6935598

[14] Roberson ED Mucke L. 100 years and counting: prospects for defeating Alzheimer's disease. Science. (2006) 314:781–4. 10.1126/science.1132813 17082448 PMC3544944

[15] Cummings J Lee G Mortsdorf T Ritter A Zhong K. Alzheimer's disease drug development pipeline: (2017). Alzheimers Dement. (2017) 3:367–84. 10.1016/j.trci.2017.05.002 29067343 PMC5651419

[16] Petersen RC. Mild cognitive impairment. Continuum. (2016) 22:404. 10.1212/CON.0000000000000313 27042901 PMC5390929

[17] Robert PH Berr C Volteau M Bertogliati-Fileau C Benoit M Guerin O. Importance of lack of interest in patients with mild cognitive impairment. Am J Geriatr Psychiatry. (2008) 16:770–6. 10.1097/JGP.0b013e31817e73db 18757769

[18] Kelley BJ Petersen RC. Alzheimer's disease and mild cognitive impairment. Neurol Clin. (2007) 25:577–609. 10.1016/j.ncl.2007.03.008 17659182 PMC2682228

[19] Petersen RC Roberts RO Knopman DS Boeve BF Geda YE Ivnik RJ. Mild cognitive impairment: ten years later. Arch Neurol. (2009) 66:1447–55. 10.1001/archneurol.2009.266 20008648 PMC3081688

[20] Rossini PM Dal Forno G. Integrated technology for evaluation of brain function and neural plasticity. Phys Med Rehabil Clin. (2004) 15:263–306. 10.1016/S1047-9651(03)00124-4 15029909

[21] Niedermeyer E da Silva FL. Electroencephalography: Basic Principles, Clinical Applications, and Related Fields. Ambler, Pennsylvania: Lippincott Williams & Wilkins. (2005).

[22] Babiloni C Lizio R Marzano N Capotosto P Soricelli A Triggiani AI. Brain neural synchronization and functional coupling in Alzheimer's disease as revealed by resting state EEG rhythms. Int J Psychophysiol. (2016) 103:88–102. 10.1016/j.ijpsycho.2015.02.008 25660305

[23] Babiloni C Lizio R Del Percio C Marzano N Soricelli A Salvatore E. Cortical sources of resting state EEG rhythms are sensitive to the progression of early stage Alzheimer's disease. J Alzheimers Dis. (2013) 34:1015–35. 10.3233/JAD-121750 23340039

[24] Cassani R Estarellas M San-Martin R Fraga FJ Falk TH. Systematic review on resting-state EEG for Alzheimer's disease diagnosis and progression assessment. Dis Markers. (2018) 2018:5174815. 10.1155/2018/5174815 30405860 PMC6200063

[25] Monllor P Cervera-Ferri A Lloret MA Esteve D Lopez B Leon JL. Electroencephalography as a non-invasive biomarker of Alzheimer's disease: a forgotten candidate to substitute CSF molecules? Int J Mol Sci. (2021) 22:10889. 10.3390/ijms221910889 34639229 PMC8509134

[26] Kwak YT. Quantitative EEG findings in different stages of Alzheimer's disease. J Clin Neurophysiol. (2006) 23:457–62. 10.1097/01.wnp.0000223453.47663.63 17016157

[27] Babiloni C Triggiani AI Lizio R Cordone S Tattoli G Bevilacqua V. Classification of single normal and Alzheimer's disease individuals from cortical sources of resting state EEG rhythms. Front Neurosci. (2016) 10:47. 10.3389/fnins.2016.00047 26941594 PMC4763025

[28] Meghdadi AH Stevanović Karić M McConnell M Rupp G Richard C Hamilton J. Resting state EEG biomarkers of cognitive decline associated with Alzheimer's disease and mild cognitive impairment. PLoS ONE. (2021) 16:e0244180. 10.1371/journal.pone.0244180 33544703 PMC7864432

[29] Van der Hiele K Vein A Reijntjes R Westendorp R Bollen E Van Buchem M. EEG correlates in the spectrum of cognitive decline. Clin Neurophysiol. (2007) 118:1931–9. 10.1016/j.clinph.2007.05.070 17604688

[30] Yan Y Zhao A Ying W Qiu Y Ding Y Wang Y. Functional connectivity alterations based on the weighted phase lag index: an exploratory electroencephalography study on Alzheimer's Disease. Curr Alzheimer Res. (2021) 18:513–22. 10.2174/1567205018666211001110824 34598666

[31] Pijnenburg YA Vd Made Y Van Walsum AVC Knol D Scheltens P Stam CJ. synchronization likelihood in mild cognitive impairment and Alzheimer's disease during a working memory task. Clin Neurophysiol. (2004) 115:1332–9. 10.1016/j.clinph.2003.12.029 15134700

[32] Jeong J EEG. dynamics in patients with Alzheimer's disease. Clin Neurophysiol. (2004) 115:1490–505. 10.1016/j.clinph.2004.01.001 15203050

[33] Abásolo D Hornero R Espino P Alvarez D Poza J. Entropy analysis of the EEG background activity in Alzheimer's disease patients. Physiol Meas. (2006) 27:241. 10.1088/0967-3334/27/3/003 16462011

[34] Liang Z Wang Y Sun X Li D Voss LJ Sleigh JW. EEG entropy measures in anesthesia. Front Comput Neurosci. (2015) 9:16. 10.3389/fncom.2015.00016 25741277 PMC4332344

[35] Mizuno T Takahashi T Cho RY Kikuchi M Murata T Takahashi K. Assessment of EEG dynamical complexity in Alzheimer's disease using multiscale entropy. Clin Neurophysiol. (2010) 121:1438–46. 10.1016/j.clinph.2010.03.025 20400371 PMC2914820

[36] Sun J Wang B Niu Y Tan Y Fan C Zhang N. Complexity analysis of EEG, MEG, and fMRI in mild cognitive impairment and Alzheimer's disease: a review. Entropy. (2020) 22:239. 10.3390/e22020239 33286013 PMC7516672

[37] Rodriguez E George N Lachaux JP Martinerie J Renault B Varela FJ. Perception's shadow: long-distance synchronization of human brain activity. Nature. (1999) 397:430–3. 10.1038/17120 9989408

[38] Jack CR Jr Albert MS Knopman DS McKhann GM Sperling RA Carrillo MC. Introduction to the recommendations from the National Institute on Aging-Alzheimer's Association workgroups on diagnostic guidelines for Alzheimer's disease. Alzheimers Dement. (2011) 7:257–62. 10.1016/j.jalz.2011.03.004 21514247 PMC3096735

[39] Xing M Tadayonnejad R MacNamara A Ajilore O DiGangi J Phan KL. Resting-state theta band connectivity and graph analysis in generalized social anxiety disorder. NeuroImage Clin. (2017) 13:24–32. 10.1016/j.nicl.2016.11.009 27920976 PMC5126152

[40] Jeong J Gore JC Peterson BS. Mutual information analysis of the EEG in patients with Alzheimer's disease. Front Neurosci. (2001) 112:827–35. 10.1016/S1388-2457(01)00513-2 11336898

[41] BaE Femir B Emek-SavaDD Güntekin B Yener GG. Increased long distance event-related gamma band connectivity in Alzheimer's disease. NeuroImage Clin. (2017) 14:580–90. 10.1016/j.nicl.2017.02.021 28367402 PMC5361871

[42] Briels CT Schoonhoven DN Stam CJ de Waal H Scheltens P Gouw AA. Reproducibility of EEG functional connectivity in Alzheimer's disease. Alzheimers Res Ther. (2020) 12:1–14. 10.1186/s13195-020-00632-3 32493476 PMC7271479

[43] Wang J Zuo X Dai Z Xia M Zhao Z Zhao X. Disrupted functional brain connectome in individuals at risk for Alzheimer's disease. Biol Psychiatry. (2013) 73:472–81. 10.1016/j.biopsych.2012.03.026 22537793

[44] Tóth B File B Boha R Kardos Z Hidasi Z Gaál ZA. EEG network connectivity changes in mild cognitive impairment Preliminary results. Int J Psychophysiol. (2014) 92:1–7. 10.1016/j.ijpsycho.2014.02.001 24508504

[45] Zeng K Wang Y Ouyang G Bian Z Wang L Li X. Complex network analysis of resting state EEG in amnestic mild cognitive impairment patients with type 2 diabetes. Front Comput Neurosci. (2015) 9:133. 10.3389/fncom.2015.00133 26578946 PMC4624867

[46] Bian Z Li Q Wang L Lu C Yin S Li X. Relative power and coherence of EEG series are related to amnestic mild cognitive impairment in diabetes. Front Aging Neurosci. (2014) 6:11. 10.3389/fnagi.2014.00011 24550827 PMC3912457

[47] Das S Puthankattil SD. Complex network analysis of MCI-AD EEG signals under cognitive and resting state. Brain Res. (2020) 1735:146743. 10.1016/j.brainres.2020.146743 32114060

[48] Sporns O Tononi G Kötter R. The human connectome: a structural description of the human brain. PLoS Comput Biol. (2005) 1:e42. 10.1371/journal.pcbi.0010042 16201007 PMC1239902

[49] Rubinov M Sporns O. Complex network measures of brain connectivity: uses and interpretations. Neuroimage. (2010) 52:1059–69. 10.1016/j.neuroimage.2009.10.003 19819337

[50] De Haan W Pijnenburg YA Strijers RL van der Made Y van der Flier WM Scheltens P. Functional neural network analysis in frontotemporal dementia and Alzheimer's disease using EEG and graph theory. BMC Neurosci. (2009) 10:1–12. 10.1186/1471-2202-10-101 19698093 PMC2736175

[51] Tijms BM Wink AM de Haan W van der Flier WM Stam CJ Scheltens P. Alzheimer's disease: connecting findings from graph theoretical studies of brain networks. Neurobiol Aging. (2013) 34:2023–36. 10.1016/j.neurobiolaging.2013.02.020 23541878

[52] Zhang B Zhang X Zhang F Li M Schwarz CG Zhang J. Characterizing topological patterns in amnestic mild cognitive impairment by quantitative water diffusivity. J Alzheimers Dis. (2015) 43:687–97. 10.3233/JAD-140882 25114082

[53] Rossini PM Miraglia F Alù F Cotelli M Ferreri F Di Iorio R. Neurophysiological hallmarks of neurodegenerative cognitive decline: the study of brain connectivity as a biomarker of early dementia. J Pers Med. (2020) 10:34. 10.3390/jpm10020034 32365890 PMC7354555

[54] Seo EH Lee DY Lee JM Park JS Sohn BK Lee DS. Whole-brain functional networks in cognitively normal, mild cognitive impairment, and Alzheimer's disease. PLoS ONE. (2013) 8:e53922. 10.1371/journal.pone.0083205 23335980 PMC3545923

[55] Vecchio F Miraglia F Marra C Quaranta D Vita MG Bramanti P. Human brain networks in cognitive decline: a graph theoretical analysis of cortical connectivity from EEG data. J Alzheimers Dis. (2014) 41:113–27. 10.3233/JAD-132087 24577480

[56] Vecchio F Miraglia F Alú F Orticoni A Judica E Cotelli M. Contribution of graph theory applied to EEG data analysis for Alzheimer's disease versus vascular dementia diagnosis. J Alzheimers Dis. (2021) 82:871–9. 10.3233/JAD-210394 34092648

[57] Youssef N Xiao S Liu M Lian H Li R Chen X. Functional brain networks in mild cognitive impairment based on resting electroencephalography signals. Front Comput Neurosci. (2021) 15:698386. 10.3389/fncom.2021.698386 34776913 PMC8579961

[58] Petersen RC Smith GE Waring SC Ivnik RJ Kokmen E Tangelos EG. Aging, memory, and mild cognitive impairment. Int Psychogeriatr. (1997) 9:65–9. 10.1017/S1041610297004717 9447429

[59] Kim JI Sunwoo MK Sohn YH Lee PH Hong JY. The MMSE and MoCA for screening cognitive impairment in less educated patients with Parkinson's disease. J Mov Disord. (2016) 9:152. 10.14802/jmd.16020 27667187 PMC5035941

[60] Welch P. The use of fast Fourier transform for the estimation of power spectra: a method based on time averaging over short, modified periodograms. IEEE Trans Audio Electroacoust. (1967) 15:70–3. 10.1109/TAU.1967.1161901

[61] Gramfort A Luessi M Larson E Engemann DA Strohmeier D Brodbeck C. MEG and EEG data analysis with MNE-python. Front Neurosci. (2013) 7:1–13. 10.3389/fnins.2013.00267 24431986 PMC3872725

[62] Costa M Goldberger AL Peng CK. Multiscale entropy analysis of biological signals. Phys Rev E. (2005) 71:021906. 10.1103/PhysRevE.71.021906 15783351

[63] Makowski D Pham T Lau ZJ Brammer JC Lespinasse F Pham H. NeuroKit2: a Python toolbox for neurophysiological signal processing. Behav Res Methods. (2021) 53:1689–96. 10.3758/s13428-020-01516-y 33528817

[64] Richman JS. Physiological time-series analysis using approximate entropy and sample entropy. Am J Physiol Heart Circ Physiol. (2000) 278:H2039–49. 10.1152/ajpheart.2000.278.6.H2039 10843903

[65] Bandt C Pompe B. Permutation entropy: a natural complexity measure for time series. Phys Rev E. (2002) 88:174102. 10.1103/PhysRevLett.88.174102 12005759

[66] Vinck M Oostenveld R Van Wingerden M Battaglia F Pennartz CM. An improved index of phase-synchronization for electrophysiological data in the presence of volume-conduction, noise and sample-size bias. Neuroimage. (2011) 55:1548–65. 10.1016/j.neuroimage.2011.01.055 21276857

[67] Bassett DS Bullmore E. Small-world brain networks. The Neuroscientist. (2006) 12:512–23. 10.1177/1073858406293182 17079517

[68] Yao Z Zhang Y Lin L Zhou Y Xu C Jiang T. Abnormal cortical networks in mild cognitive impairment and Alzheimer's disease. PLoS Comput Biol. (2010) 6:e1001006. 10.1371/journal.pcbi.1001006 21124954 PMC2987916

[69] Hagberg A Schult D Swart P. Exploring network structure, dynamics, and function using NetworkX. In: Proc 7th Python in Science Conf . Pasadena, CA. (2008) p. 11–15.

[70] Latora V Marchiori M. Efficient behavior of small-world networks. Phys Rev Lett. (2001) 87:198701. 10.1103/PhysRevLett.87.198701 11690461

[71] Strang A Haynes O Cahill ND Narayan DA. Generalized relationships between characteristic path length, efficiency, clustering coefficients, and density. Soc Netw Anal Min. (2018) 8:1–6. 10.1007/s13278-018-0492-3

[72] Achard S Bullmore E. Efficiency and cost of economical brain functional networks. PLoS Comput Biol. (2007) 3:e17. 10.1371/journal.pcbi.0030017 17274684 PMC1794324

[73] Kim SE Kim HS Kwak Y Ahn MH Choi KM Min BK. Neurodynamic correlates for the cross-frequency coupled transcranial alternating current stimulation during working memory performance. Front Neurosci. (2022) 16:1013691. 10.3389/fnins.2022.1013691 36263365 PMC9574066

[74] Watts DJ Strogatz SH. Collective dynamics of “small-world” networks. Nature. (1998) 393:440–2. 10.1038/30918 9623998

[75] Humphries MD Gurney K. Network “small-world-ness”: a quantitative method for determining canonical network equivalence. PLoS ONE. (2008) 3:e0002051. 10.1371/journal.pone.0002051 18446219 PMC2323569

[76] Benjamini Y Hochberg Y. Controlling the false discovery rate: a practical and powerful approach to multiple testing. J R Stat Soc B. (1995) 57:289–300. 10.1111/j.2517-6161.1995.tb02031.x

[77] Fröhlich S Kutz DF Müller K Voelcker-Rehage C. Characteristics of resting state EEG power in 80+-year-olds of different cognitive status. Front Aging Neurosci. (2021) 13:675689. 10.3389/fnagi.2021.675689 34456708 PMC8387136

[78] Morison G Tieges Z Kilborn K. Multiscale permutation entropy analysis of the EEG in early stage Alzheimer's patients. In: 2012 IEEE-EMBS Conference on Biomedical Engineering and Sciences. Langkawi. (2012) p. 805–9.

[79] Afshari S Jalili M. Directed functional networks in Alzheimer's Disease: disruption of global and local connectivity measures. IEEE J Biomed Health Inform. (2017) 21:949–55. 10.1109/JBHI.2016.2578954 27305688

[80] Sanabria-Diaz G Martinez-Montes E Melie-Garcia L Initiative ADN. Glucose metabolism during resting state reveals abnormal brain networks organization in the Alzheimer's disease and mild cognitive impairment. PLoS ONE. (2013) 8:e68860. 10.1371/journal.pone.0068860 23894356 PMC3720883

[81] Button KS Ioannidis JP Mokrysz C Nosek BA Flint J Robinson ES. Power failure: why small sample size undermines the reliability of neuroscience. Nat Rev Neurosci. (2013) 14:365–76. 10.1038/nrn3475 23571845

[82] Han Y Wang K Jia J Wu W. Changes of EEG spectra and functional connectivity during an object-location memory task in Alzheimer's disease. Front Behav Neurosci. (2017) 11:107. 10.3389/fnbeh.2017.00107 28620287 PMC5449767

